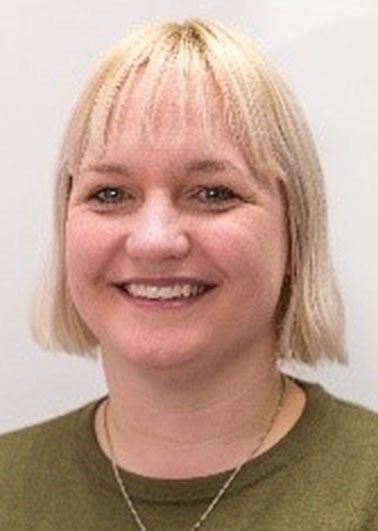# Methodologies for “Wiring” Redox Proteins/Enzymes to Electrode Surfaces

**DOI:** 10.1002/chem.201800750

**Published:** 2018-06-06

**Authors:** Nicholas D. J. Yates, Martin A. Fascione, Alison Parkin

**Affiliations:** ^1^ Department of Chemistry University of York Heslington Road York YO10 5DD UK

**Keywords:** electrochemistry, immobilization, protein modifications, proteins, surface chemistry

## Abstract

The immobilization of redox proteins or enzymes onto conductive surfaces has application in the analysis of biological processes, the fabrication of biosensors, and in the development of green technologies and biochemical synthetic approaches. This review evaluates the methods through which redox proteins can be attached to electrode surfaces in a “wired” configuration, that is, one that facilitates direct electron transfer. The feasibility of simple electroactive adsorption onto a range of electrode surfaces is illustrated, with a highlight on the recent advances that have been achieved in biotechnological device construction using carbon materials and metal oxides. The covalent crosslinking strategies commonly used for the modification and biofunctionalization of electrode surfaces are also evaluated. Recent innovations in harnessing chemical biology methods for electrically wiring redox biology to surfaces are emphasized.

## 1 Introduction to biological redox chemistry

**Figure 1:**
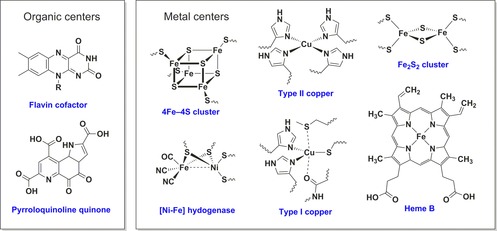
Examples of the diverse range of redox centers utilized in redox proteins and enzymes.

**Figure 2:**
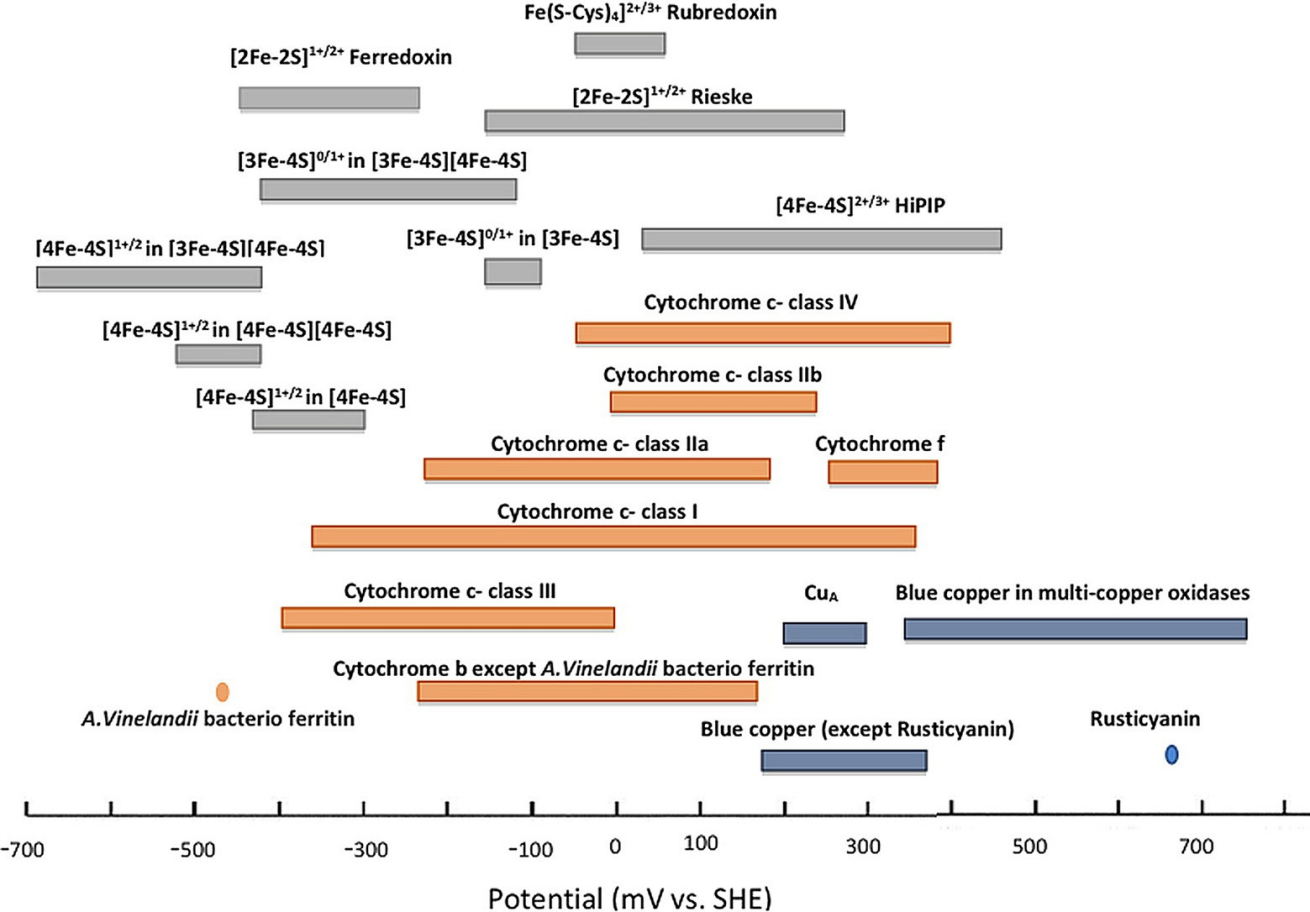
The voltage range spanned by biological redox centers. Reproduced with permission from ref. [1].

The proteins that facilitate biological electron transfer processes are referred to as “redox proteins.” These molecules play essential roles in processes ranging from photosynthesis to respiration, from bioluminescence to nitrogen fixation, and from nucleic acid biosynthesis to apoptosis.^1^, ^2^ The thermodynamics and kinetics of the biological electron transfer reactions are determined by the nature of the redox centers within the participating proteins. These redox‐active centers can be either organic cofactors (e.g., quinones and flavins)^3^ or metal centers (e.g., iron sulfur clusters and Cu sites),^1^ as exemplified by Figure 1. Redox enzymes are a subset of redox‐active proteins that catalyze the oxidation or reduction of substrate molecules at a redox‐active center. The approximately 1.5 V potential window which such biological redox centers span (see Figure 2) is wider than the thermodynamic stability window of water, since proton reduction to hydrogen (*E*(2H^+^/H_2_)=−0.41 V at pH 7) and water oxidation to oxygen (*E*(O_2_/H_2_O)= +0.82 V at pH 7) are both processes which have been occurring in biology for millennia.^4^ Recent work on azurin, a single‐copper electron‐transfer protein, has elegantly demonstrated how the reduction potential of biological redox centers is tuned by the interplay between both the redox center architecture and the surrounding protein structure.^5^, ^6^

Research interest in biological redox chemistry is inspired by more than just an academic curiosity in understanding the biochemical reactions of life. Enzymes play an essential role in the production of biofuels, and redox‐active metalloenzymes play a particularly vital role in hydrogen generation,^7^, ^8^, ^9^ methane production,^10^ and a recently discovered role in cellulose breakdown.^11^ Other redox‐enzyme based applications range from the development of novel biocatalysts for solving challenging synthetic problems,^12^, ^13^ to the sequestering of atmospheric CO_2_.^14^, ^15^ As both redox‐active proteins and enzymes can be used to elicit an electronic response from a biological stimulus, there is also a vast range of literature exploring the use of such molecules for developing new sensor technologies.^16^ One of the most famous examples is the blood glucose sensor, a device that helps billions of people worldwide by monitoring the concentration of glucose in the bloodstream through the electrochemical response of glucose oxidase.^17^, ^18^

**Figure 3:**
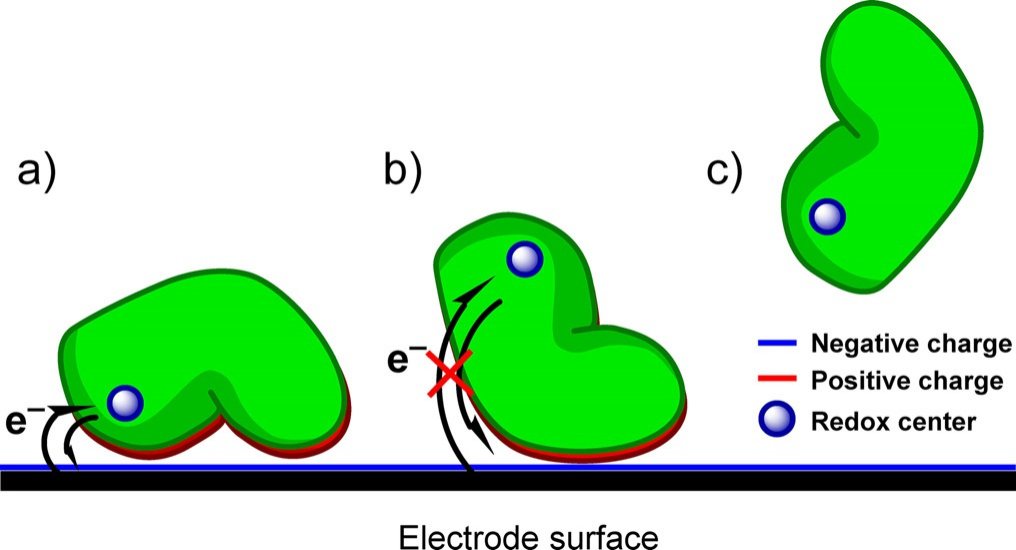
Non‐specific protein adsorption outcomes. a) Electrostatic attraction of oppositely charged protein residues and electrode surface facilitates the immobilization of the protein in an electroactive orientation, facilitating direct electron transfer between a redox center and the electrode. b) Protein becomes immobilized in an orientation that does not facilitate direct electron transfer. c) Protein does not adsorb to the electrode surface.

This Review focuses on strategies for stably attaching proteins and enzymes onto surfaces. Regardless of whether proteins or enzymes are redox‐active or not, the analytical study and commercial utilization of such biomolecules is aided by such immobilization methodologies. For example, surface plasmon resonance (SPR) detection of drug molecule binding is inherently reliant on the attachment of proteins or enzymes onto sensor chips^19^ or nanoparticles.^20^ In industrial catalysis, the localization of enzymes on the surface of a solid support can also help overcome high operation costs, improving the ease of separation of enzyme from product, the lifetime and reusability of the enzyme, and potentially enhancing the thermostability.^21^ In the case of redox proteins and enzymes, immobilization onto a conducting surface provides a route for the delivery or removal of electrons (Figure 3). Such a “wired” biomolecule–surface configuration can either be utilized in electroanalytical measurements that probe the biological redox process, or for constructing electrodes for biotechnological applications such as medical bio‐sensing,^22^ solar fuel production,^23^, ^24^ or cofactor regeneration systems.^25^

## 2 Electroactive protein adsorption onto unmodified conducting surfaces

The orientation of redox proteins or enzymes onto electrode surfaces in a so‐called “electroactive” configuration, that is, one that permits direct electron transfer between the surface and the biomolecule, is a prerequisite for protein film electrochemistry, often referred to as PFE.^16^, ^18^, ^26^, ^27^, ^28^, ^29^ This technique quantifies the thermodynamic and kinetic parameters of the electrochemical reactions of redox proteins and enzymes which form a “film” on the surface of the working electrode that is interrogated using a standard three‐electrode electrochemical setup.^16^, ^18^, ^26^, ^27^, ^28^, ^29^ The wealth of detailed mechanistic PFE studies conducted using a wide range of different proteins or enzymes directly adsorbed onto electrodes demonstrates the feasibility of immobilizing such redox‐active macromolecules onto solid surfaces through noncovalent interactions.^16^, ^18^, ^26^, ^27^, ^28^, ^29^ When successful, such an immobilization strategy clearly represents the simplest approach for achieving electroactive films of redox protein or enzyme.

In living systems, the exchange of electrons between soluble redox proteins is dependent on the two proteins “docking” so that the electron‐donor and electron‐acceptor redox centers are brought into close enough approach to facilitate rapid, direct electron transfer.^30^ These interactions are often mediated by areas of complementary polarity on the donor and acceptor proteins.^30^ Thus, a simple model for understanding successful direct electroactive protein adsorption onto electrode surfaces is to envisage the electrode surface polarity complementing a region of oppositely charged residues on the protein surface that is proximal to the electron entry/exit redox center (Figure 3).^31^ This means that direct electron transfer between redox proteins and electrode surfaces is most easily achieved when the electron entry/exit redox center is close to the protein surface.^32^ The adsorption and orientation of proteins onto surfaces can be influenced by the solution electrolyte conditions. As described below, ionic strength and pH are both important variables, and the entropically disfavored process of adsorption is also favored at lower temperatures.^29^

Detailed work by Harry Gray and co‐workers has demonstrated that electrons tunnel through the protein structure which separates electron‐donor and electron‐acceptor partner redox‐active centers, and therefore distance plays a crucial role in determining the rate of electron transfer.^33^, ^34^ A helpful rule‐of‐thumb provided by Dutton and co‐workers is that within metalloenzyme structures a tunneling distance of less than 14 Å between redox active sites appears to support electron transfer rates that are sufficiently fast to avoid limiting the rate of redox catalysis.^31^ Ideally, all protein or enzyme molecules would therefore orient on the electrode with the same sub‐14 Å distance between the redox‐active center and the conducting surface. However, in some cases the electrochemical response of otherwise identical protein molecules differs, and this has been attributed to a “dispersion” in protein/enzyme orientation.^7^, ^29^, ^35^

Redox proteins/enzymes can also become adsorbed onto surfaces in configurations which do not facilitate direct electron transfer at all, as illustrated in Figure 3. Alternatively, the biomolecules may remain in solution, with the slow rate of diffusion of these macromolecules impeding solution electrochemistry, and electron transfer to the electrode instead relying on the introduction of redox mediators. Such mediated bioelectrochemistry is extremely useful in sensor development,^36^, ^37^, ^38^ and careful design of electron‐transfer polymer gels can even permit simultaneous entrapment of enzymes on the electrode and modification of the reactivity. For example, a H_2_ enzyme was recently made functional in O_2_‐saturated solution through use of a viologen‐polymer net.^39^ However, as noted above, herein we focus our attention on protein/enzyme–electrode immobilization strategies that permit direct, unmediated electron transfer.

No tools are currently available to predict the likelihood that a redox protein or enzyme of interest will become adsorbed in an electroactive configuration on a solid support, and screening for a successful protein–surface combination remains an empirical process.^29^ The electrode surfaces most commonly used for electroactive protein/enzyme electrode immobilization are briefly reviewed below.

## 3 Carbon electrodes

### 3.1 Carbon bulk materials

**Figure 4:**
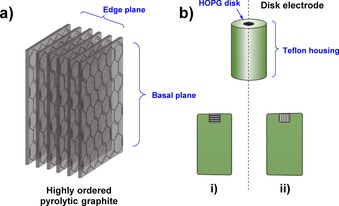
a) The edge‐ and basal planes of highly ordered pyrolytic graphite (HOPG). b) The different potential configurations of HOPG in disk electrodes, either: i) with the basal plane exposed, or ii) the edge plane (often denoted pyrolytic graphite edge or “PGE” electrodes).

Carbon is an extremely popular material for constructing electrodes for the electrochemical interrogation of small molecules.^40^ As a highly conductive allotrope of carbon, graphite electrodes are common.^40^ Either pyrolytic graphite edge (PGE) or basal plane graphite (BPG) electrodes can be fabricated from cutting highly ordered pyrolytic graphite (HOPG) substrate in perpendicular directions, across or parallel to the graphite sheets, respectively (Figure 4).^41^

**Figure 5:**
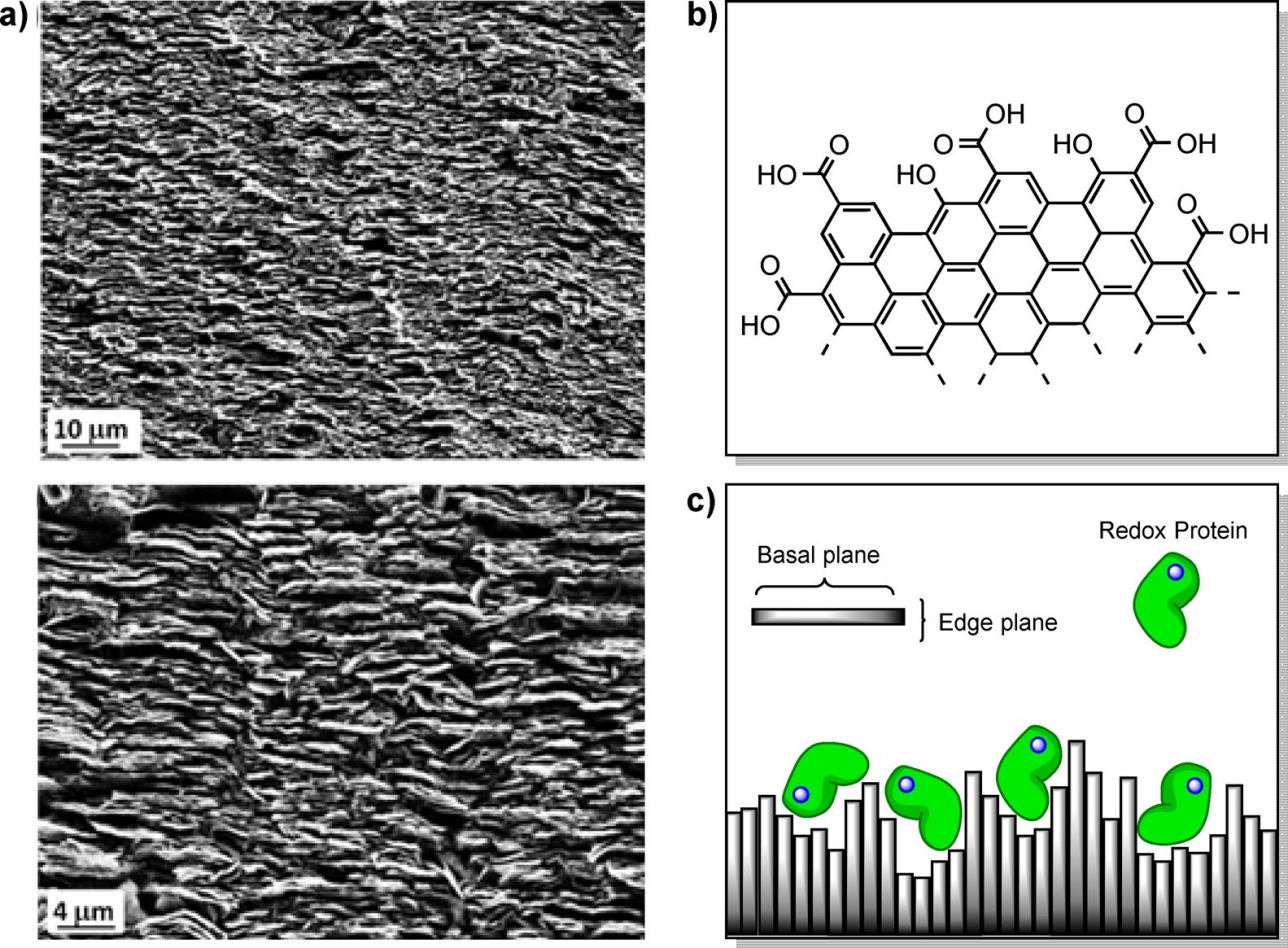
a) SEM images of the rough surface topology of PGE adapted with permission from ref. 50. b) The chemical groups presented at the edge plane of HOPG. c) The facilitation of electroactive adsorption of redox proteins onto PGE by the rough topology of the surface.

For PFE, PGE has proved to be the most successful carbon electrode material for the electroactive adsorption of redox proteins and enzymes.^16^, ^18^, ^26^, ^27^, ^28^, ^29^, ^42^, ^43^ This has been attributed to the PGE surface components, including a diverse range of aromatic, hydrophilic (i.e. phenolic), and carboxylate functionalities that are present as defects on the edge plane (Figure 5), yielding a generally negatively charged surface that will electrostatically attract regions of complementary positive polarity on the protein surface.^44^, ^45^ Electrode surface polishing/abrasion processes using emery paper or pastes of diamond or alumina are often used to actively increase the surface roughness and thus increase the number of defect sites.^29^ Alumina and diamond polishing materials can remain on the electrode surface even after rinsing and sonicating the electrodes, so it is also possible that the presence of polishing materials contributes to the performance of PGE electrodes.^43^

The combination of the chemical heterogeneity and the topological roughness of the PGE surface has also been credited with making it particularly suitable for electroactive protein/enzyme immobilization.^42^ The chemical heterogeneity allows multiple and varied favorable contacts to be made between the protein and the electrode surface.^29^ The roughness of the surface can ensure that a range of immobilized protein orientations are electroactive,^46^ as even if the face through which the protein is adsorbed to the electrode surface is distant from an electron entry/exit site, rapid electron transfer may still be feasible because this site is close to another part of the electrode surface (Figure 5).^29^ Such orientational flexibility may also explain a statistical variation in the electrochemical reaction parameters. For example, in H_2_‐enzyme voltammetry modelling studies, the need to include a range of different interfacial electron‐transfer kinetic rate constants in the calculations is attributed to dispersion in the distance between the electrode surface and the electron entry/exit site in the protein.^16^, ^35^, ^47^ In studies on azurin, variations in the apparent midpoint potential of the biological electron transfer were attributed to different protein–surface orientations/environments.^48^, ^49^

**Figure 6:**
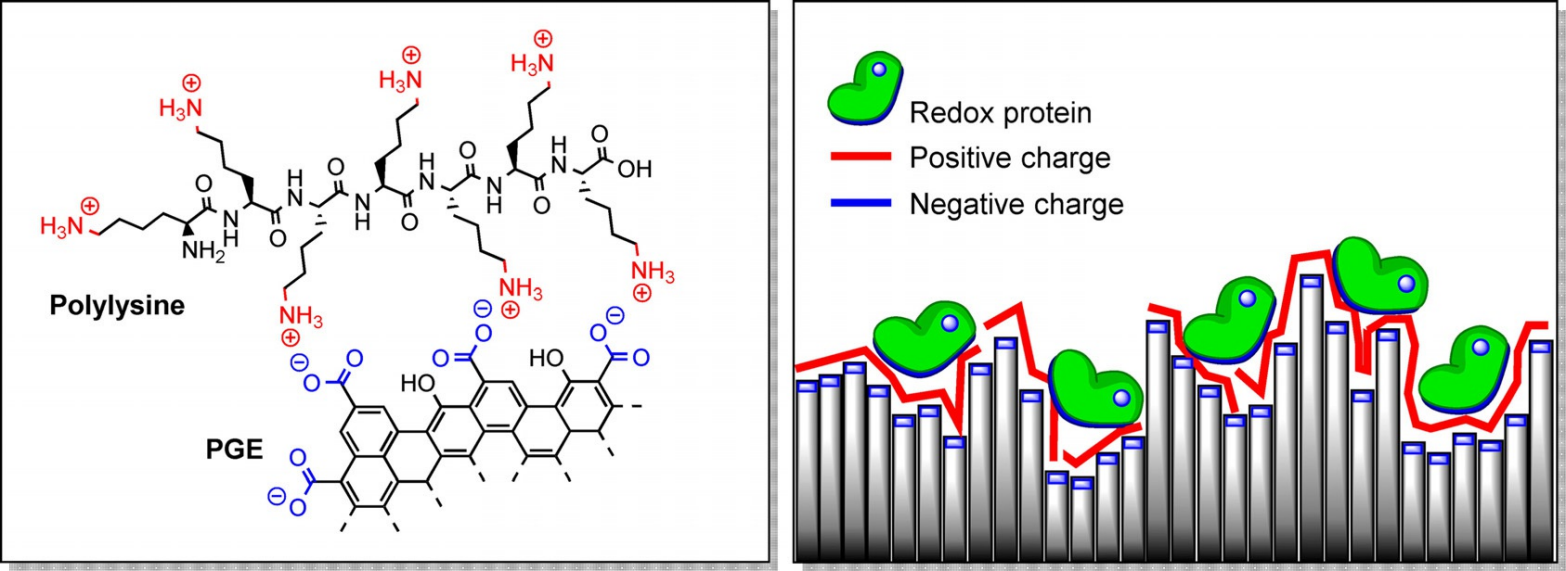
Left: the interaction of polycationic species such as polylysine with negatively charged PGE surfaces. Right: the formation of ternary salt bridges between negatively charged protein surfaces and negatively charged electrode surfaces using polycationic species can facilitate the adsorption of negatively charged proteins.

In cases where the electron entry/exit site of the protein or enzyme is located within a region of negative charge, the polarity of the PGE surface may not help facilitate electroactive adsorption, and may instead promote the desorption of adsorbed proteins.^45^, ^51^ Alleviating the electrostatic repulsion between protein and electrode can be achieved through mild acidification, or through the co‐adsorption of protein with polycationic hydrophilic compounds such as aminocyclitols,^32^ polylysine,^32^ polymyxin^16^, ^29^, ^51^ or polyethyleneimine.^45^, ^51^ Polycationic species have an affinity for the PGE surface, and are thought to mediate protein adsorption through the formation of ternary salt bridges between areas of negative charge on the protein and electrode surface (Figure 6).^29^, ^32^

Aside from PGE, other carbon surfaces have also proved successful for producing electroactive films of redox proteins or enzymes. Carbon felt is comprised of an amorphous tangle of smooth carbon fibers.^52^ The high surface area, high conductivity, large void spaces and low cost of this material make it suitable for application in redox‐enzyme biofuel devices.^52^ Carbon felt electrodes of small geometric surface area can accommodate and directly exchange electrons with large quantities of enzymes, with a diiron hydrogenase used in a bio‐H_2_ device.^53^ Such porous materials can be less useful in mechanistic studies of redox enzymes since the diffusion rates of substrate, product, or inhibitor through the material may limit the rate of reactivity. This would mean that electrochemical current cannot be used to monitor the inherent maximum turnover rate of the enzyme. However, in enzyme fuel cell developments, where the focus is to maximize the enzyme current per unit surface area, such porous materials are very useful, and have enabled order‐of‐magnitude power increases.^54^

In solution‐state electrochemical studies of small redox‐active molecules, common carbon‐based electrode substrates include boron‐doped diamond (BDD) and glassy carbon (GC).^55^ BDD consists of diamond in which approximately one atom in a thousand has been replaced by boron, giving the material *p*‐type semiconductive properties and yielding the hardest carbon material used for electrodes.^41^ The very low capacitance of BDD minimizes background current, effectively enhancing the sensitivity of the electrochemical measurement.^41^ However, BDD is not widely utilized in PFE, presumably indicating that the surface electrostatics do not facilitate protein adsorption. The structure of GC consists of interwoven graphite ribbons, reminiscent of three‐dimensional chainmail.^41^ GC is much harder than HOPG, and contains hydrophobic basal‐like and hydrophilic edge‐like regions within the same plane. This complex surface can facilitate the adsorption of some proteins onto the bare GC surface,^16^, ^56^ but much of the recent literature using GC electrodes for direct immobilization of redox proteins describes the functionalization of the GC surface with nanomaterials, such as carbon nanotubes (CNTs),^57^, ^58^, ^59^, ^60^ carbon black,^61^ and even silicon dioxide nanoparticles.^62^

### 3.2 Carbon nanomaterials

There are two classes of CNT: single‐wall carbon‐nanotubes (SWCNTs) and multi‐wall carbon nanotubes (MWCNTs).^63^, ^64^ SWCNTs have a cylindrical nanostructure, and can be thought of as a single graphite sheet rolled up into a tube,^63^ whereas MWCNTs comprise several layers of SWCNTs concentrically arranged like rings in a tree trunk.^63^ The ability of CNTs to mediate direct ET is attributed to the combination of high surface area, high conductivity, and the polarities of the surfaces they present; the side walls of CNT likely have properties similar to those of the basal plane of HOPG, whereas the ends of the tubes likely have properties akin to PGE.^63^, ^65^ The walls of these nanotubes are capable of forming strong π–π interactions to small molecule species, such as pyrene.^64^

There are a variety of methods for structuring CNT/redox protein assemblies on electrode surfaces, and these methods have been comprehensively reviewed.^63^, ^66^, ^67^ Simple approaches include the evaporation of a droplet of redox protein/CNT dispersion onto a GC electrode surface, followed by the addition of a small amount of Nafion membrane to act a binding agent,^60^ or the filling of microcavities in the bulk electrode surface with CNTs.^68^ Such methods have resulted in facile direct ET being established between the electrode surface and a range of proteins, including hemoglobin,^60^ horseradish peroxidase^60^ and, remarkably, glucose oxidase;^60^, ^68^ a protein for which the establishment of direct ET is infamously difficult owing to the coenzyme flavin adenine dinucleotide (FAD) unit of GOx being deeply embedded within the protein structure.^69^ More advanced techniques, such as the construction of “CNT forests” (i.e., short SWCNTs arranged orthogonally to an electrode surface by self‐assembly^70^) provide high surface area assemblies into which redox proteins can be spontaneously incorporated,^65^ or atop which redox enzymes can be covalently wired.^71^

Graphene can be formulated as a highly conductive carbon nanomaterial, that can also be used to make electrode surfaces amenable for PFE. The attachment of graphene to a supporting electrode can be achieved through simple electrode treatments, such as the application of graphene suspensions to GC, which promotes the formation of a stable thin film owing to π–π stacking interactions.^72^ Alternatively, composite mixtures of chitosan and graphene can be applied to carbon electrode surfaces as thin films which promote the physisorption of redox proteins.^73^, ^74^, ^75^, ^76^ A review of the uses of graphene in electrochemical sensors and biosensors has been compiled by Shao et al.^77^

The functionalization of electrode surfaces with high conductivity carbon black (CB) nanomaterials, such as Ketjen Black powder,^78^ can also promote direct ET to redox proteins or enzymes.^78^, ^79^ The affinity between CB and protein surfaces has been attributed to hydrophobic–hydrophobic interactions, high porosity, and high surface‐area‐to‐volume ratio.^80^ The electroactive immobilization procedure is often performed by evaporation of suspension/slurries of CB particles onto carbon electrodes.^61^, ^78^, ^79^, ^81^ More complex hybrid bio‐synthetic catalytic systems can be generated by combining CB particles with redox enzymes and other nanoparticles. For example, Matteo Duca and co‐workers showed that a nitrate reductase from *E. coli* could be immobilized onto carbon black, and the co‐deposition onto a PGE surface of these bio‐modified particles along with Pt or Rh nanoparticles yielded a system capable of the electrocatalysis of nitrate to ammonia at neutral pH.^82^ In the absence of enzyme, the slow reduction of nitrate by the noble metal catalysts alone significantly limited the rate of denitrification, whereas the enzyme‐containing system may be applicable for wastewater treatment.^82^

### 3.3 Metal oxide semiconductors

Electrodes constructed of metal oxide semiconductors have become increasingly important in both PFE studies and metalloenzyme biotechnological device development. In particular, n‐type metal oxide semiconductors such as TiO_2_,^83^ indium tin oxide (ITO)^84^ and CdS^85^ were used for solar fuel applications^24^ and NADH recycling.^25^ TiO_2_ electrode surfaces are rough, porous structures consisting of aggregated nanoparticles.^24^ The CdS surface topology is similar, comprising a highly porous three‐dimensional network of CdS sheets.^24^, ^46^ ITO electrodes with porous architectures suitable for redox‐protein immobilization can also be constructed^84^, ^86^ and, along with PGE^24^, ^45^ and TiO_2_,^87^, ^88^ present negatively charged oxide functionalities for adsorbing protein or enzyme molecules at neutral pH.^43^, ^89^ The rough/porous nature of these electrode materials is thought to aid in electroactive enzyme immobilization, as described for PGE.^46^, ^86^ Indeed, PFE of a H_2_‐producing [FeFe]‐hydrogenase from *Clostridium acetobutylicum* was recently demonstrated using a TiO_2_ electrode,^90^, ^91^ whereas previously immobilization of [FeFe]‐hydrogenases on native electrode surfaces had only been achieved using rough carbon electrode substrates, such as PGE^92^, ^93^ or carbon felt.^53^

**Figure 7:**
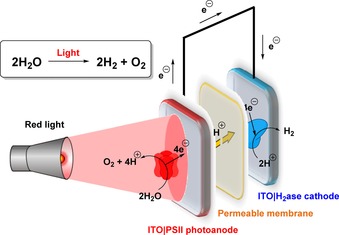
The water‐splitting photoelectrochemical cell developed by Erwin Reisner and co‐workers, utilizing photosystem II and hydrogenase enzymes immobilized on hierarchically structured ITO electrodes.^84^

Unlike PGE, ITO is transparent and the porosity of such metal oxide electrode surfaces is also readily tunable.^84^, ^86^ An especially high‐surface‐area hierarchically structured ITO electrode with a microporous inverse opal architecture and a mesoporous skeleton was recently developed by Reisner and co‐workers.^84^ Immobilization of high quantities of the enzymes photosystem II and a [NiFeSe]‐hydrogenase onto a photoanode and a cathode, respectively, yielded a photoelectrochemical solar‐water‐splitting enzyme cell (Figure 7).^84^ This device is capable of yielding a light‐to‐hydrogen conversion efficiency of as much as 5.4 %.^84^ Alternatively, using photosystem I, cytochrome c and human sulfite oxidase, Lisdat and co‐workers have demonstrated the possibility of using ITO as a support for light‐driven bio‐sensing redox enzyme devices.^94^

As with PGE, nonspecific adsorption of protein to a semiconductor can be facilitated by considering the effect of pH. For example, the isoelectric point (pI) of a TiO_2_ surface was found to be 6.2,^95^ whereas the pI values of a carbon monoxide dehydrogenase^96^ and a [NiFeSe]‐hydrogenase^83^ were found to be 5.5 and 5.4, respectively. Both enzymes could be adsorbed to TiO_2_ nanoparticles at pH 6,^83^, ^97^, ^98^ and this has been rationalized by considering that under these conditions the net surface charge of the enzymes is negative whereas that of the TiO_2_ is positive. Similarly, the work of Emmanuel Topoglidis and co‐workers^95^ has shown that the adsorption to TiO_2_ of cytochrome c and hemoglobin was greater at pH 7 than at pH 6.^95^ Likewise, this was explained by considering that at pH <7.5, the proteins would be positively charged whereas the TiO_2_ surface is negatively charged at pH 7 but not at pH 6.^95^

## 4 Common electrode functionalization strategies to promote electroactive surface adsorption

In this section we outline surface functionalization strategies that make electrode surfaces amenable to electroactive redox‐protein and redox‐enzyme adsorption. The general merit of all such electrode modification strategies is that they do not require changes to be made to the protein structure. Instead, the surface–protein interactions should ideally mimic those which underpin electron exchange between the biological molecule and its redox partner(s) in vivo. Covalent bonding strategies that aim to make single, site‐specific linkages between electrodes and proteins or enzymes will be discussed in Section 5.

### 4.1 Thiol self‐assembled monolayers on gold

**Figure 8:**
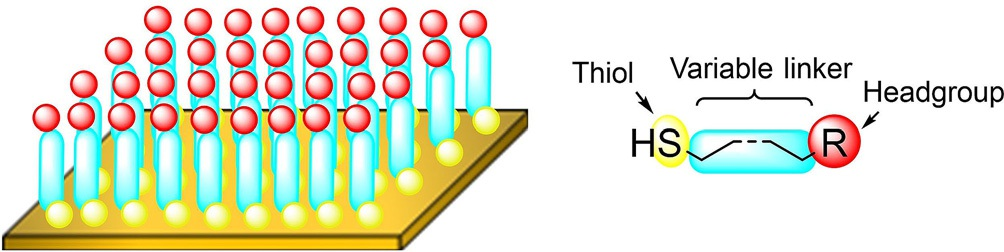
Left: depiction of an alkanethiol SAM on a gold surface, and right: the generic structure of alkanethiols used in SAM construction.

A significant amount of literature describes the immobilization of redox proteins onto surface‐modified gold nanoparticles and surface‐modified macroscopic gold surfaces.^43^ The requirement for surface modification does not arise because proteins cannot bind to gold surfaces; computational evidence suggests the alcohol moieties of serine and threonine amino acid residues can bind to crystalline Au (111) surfaces.^99^ The problem is that such interactions can induce protein unfolding.^43^ The functionalization of gold surfaces with alkanethiol based self‐assembled monolayers (SAMs) is thus common practice as it offers the dual opportunity to both mask the gold atoms^100^ and present a reactive headgroup into solution that will induce orientation of the protein in an electroactive configuration (Figure 8).^101^, ^102^, ^103^, ^104^, ^105^, ^106^, ^107^, ^108^, ^109^

SAM formation is generally achieved by immersing a clean gold substrate into a dilute solution of the desired thiol in ethanol, whereupon the thiol functionalities chemisorb to the gold, spontaneously forming S−Au bonds.^110^ The “self‐assembled” nature of the monolayer arises owing to the hydrophobic effect which drives the spontaneous vertical alignment of the alkane chains, yielding a uniform monolayer of densely packed alkanethiols (Figure 8).^111^ The gold cleaning process is necessary to remove any oxide coating and/or adsorbed organic moieties on the gold surface.^112^

Metals other than gold also form strong‐enough thiol bonds to enable alkanethiol SAM formation. This is relatively trivial for palladium, silver, and mercury that, like gold, do not form stable oxide layers.^113^, ^114^ However, it is more challenging to form high‐quality SAMs on copper,^113^ and accordingly such surface modifications are more poorly understood than those constructed on other coinage metals.^111^

The biggest limitation for using gold‐thiol based SAM systems in redox protein/enzyme electrochemical applications is that they have a limited electrode potential window over which they are stable. This window has been reported as between −0.9 and +1.0 V versus standard hydrogen electrode (SHE) at ambient temperature,^112^, ^115^ but a more conservative estimate further limits this range to between −0.4 and +0.6 V versus SHE.^43^ At a sufficiently negative potential, reduction of the gold‐thiol bond causes the SAM to detach from the surface, whereas over‐oxidization leads to SAM detachment attributed to the generation of sulfur oxides.^43^ This inherent SAM redox activity prevents the use of gold‐thiols in some bioelectrochemical applications,^43^ for example the construction of enzymatic CO_2_ reduction or H_2_O oxidation systems. SAMs also often have poor long‐term storage stability, owing to air‐induced oxidation of the metal‐thiolate bond.^112^ As exemplified below, this has not prevented the use of Au‐SAMs in a significant number of analytical bioelectrochemical studies, but potentially introduces the requirement for more stable electrode modification routes for the development of commercial technological devices.

#### 4.1.1 Single‐component SAMs on gold

Alkanethiol SAMs are frequently used to tailor the polarity of a metal electrode to complement that of the target protein, mediating immobilization through non‐specific interactions, as described in Section 2.^102^, ^103^, ^104^, ^105^, ^106^, ^107^, ^108^, ^109^, ^114^ Azurin, a blue type‐I copper protein, has been immobilized as monolayers or submonolayers using simple SAMs of different length, such as pentanethiol^103^ and decanethiol.^102^ Such non‐functionalized (i.e. alkane headgroup) alkanethiols are thought to facilitate direct electron transfer between a gold electrode and azurin because the protein has a patch of hydrophobic surface residues proximal to the redox‐active copper center.^116^, ^117^ The stability of azurin on such alkanethiol SAMs has been put to particularly good use in the quantification of kinetic and thermodynamic dispersion, through the coupling of fluorescence monitoring of the copper redox state with electrochemical control of the redox potential.^118^, ^119^

The immobilization of proteins which interact well with negatively charged PGE electrodes has been achieved through the use of carboxylic‐acid‐terminated SAMs.^105^, ^106^, ^107^, ^108^, ^109^, ^120^ This has been probed in detail using cytochrome c, a protein thought to transfer electrons through interaction with redox partners that are attracted to the positively charged surface lysine moieties close to the redox‐active haem group.^29^, ^37^, ^121^ When a SAM with SO_3_H headgroups was used instead of a COOH‐terminated SAM, electroactive electrode immobilization was still achieved.^122^ Cytochrome c has also been used in experiments to probe the impact of alkane chain length on the rate of electron transfer, *k*_ET_, between a gold electrode and a protein sitting atop a SAM. When shorter alkanethiols (≤6 carbon atoms) are used, *k*_ET_ is independent of the alkane chain length, indicating that the electrochemistry is reporting on the inherent maximum rate of the Fe^3+^ + 1 e^−^ $\vec{\leftarrow }$ Fe^2+^ biological redox process of interest.^123^, ^124^, ^125^ However, *k*_ET_ decreases exponentially with the length of the alkanethiol when molecules of more than nine carbon atoms are used.^123^, ^124^, ^125^ This indicates that the tunneling of the electron through the SAM has become the rate‐limiting step in electron transfer.^123^, ^124^, ^125^

For redox proteins bearing negative surface charges close to the electron entry/exit site, such as plastocyanin or ferredoxins,^32^ amino‐terminated SAMs can support direct electron transfer in a similar way.^37^ Alternatively, as with PGE (Figure 6), the treatment of acid‐terminated alkanethiol SAMs with poly‐l‐lysine allows for electroactive immobilization of negatively charged proteins such as cytochrome b5,^126^ avidin,^127^ and glucose oxidase,^128^ with the cationic poly‐amine again acting as an electrostatic “glue” between the negative protein and SAM surfaces.^126^

Alkanethiols are not the only molecules which can be used for the formation of SAMs that support electron transfer to an immobilized redox protein or enzyme. Short peptides have been used to form SAMs that permit the electrochemical assay of cytochrome b562 from *E. coli* 129 and a methane monooxygenase from *Methylococcus capsulatus*.^130^

#### 4.1.2 Multicomponent SAMs on gold

Mixing two or more different alkanethiol molecules together enables the formation of multicomponent SAMs. For example, myoglobin has been stabilized by forming a multicomponent SAM using alkanethiols with OH headgroups and alkanethiols with COOH headgroups.^104^ In certain electroanalytical applications, mixed SAM systems may prove superior to single‐component SAM modifications. The standard rate constant for electron transfer, *k*_ET_, to cytochrome c immobilized on a multicomponent SAM of composition 8:2 mercaptoundecanoic acid (MUA) to decanethiol was about five times greater than that on a single‐component SAM of MUA at pH 7.^122^, ^131^ This was attributed to the notion that deprotonation of the headgroups of a SAM formed from just COOH‐terminated alkanethiols introduces such a high concentration of negative charge on the surface of the electrode that immobilized proteins are induced to adopt an orientation that is not optimized for rapid electron transfer.^122^

**Figure 9:**
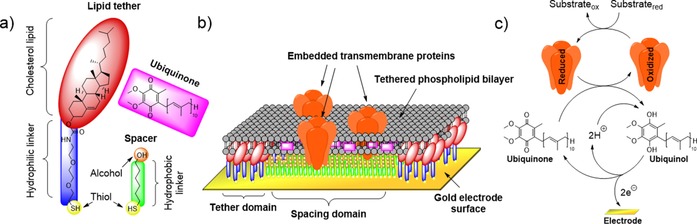
Tethered bilayer lipid membrane on gold electrode for the immobilization of membrane‐bound redox proteins. a) Structures of the components used in tethered bilayer lipid membrane assembly: lipid tethers, spacer units, and quinone‐type molecules. b) Structure of a tethered bilayer lipid membrane on a gold electrode, including embedded transmembrane proteins. c) Mediation of electron transfer by quinone‐type molecules.^132^, ^133^, ^134^

In a particularly elegant example of biological mimicry, the incorporation of further self‐assembling layers on top of multicomponent SAMs can be used to fabricate structures that mimic biological membranes (Figure 9).^132^, ^133^ Such electrode‐confined tethered bilayer lipid membranes are constructed by first creating a multicomponent SAM using a mixture of specially designed lipid tethers and small alkanethiol molecules, such as 6‐mercaptohexanol (Figure 9).^132^, ^133^ Owing to the mismatches in chain length and polarity between these two species, they form nanoscale phase‐separated domains on the gold surface. The lipid tethers bind to the electrode surface through an Au‐S bond, while their headgroups (often cholesterol lipids) induce the self‐assembly of phospholipid layers on top of them. Phospholipid bilayers are formed to span across the alkanethiol spacer domains that sit between the lipid tether domains, and transmembrane proteins can be embedded into these bilayer regions and electrochemically interrogated, often through the mediation of electron transfer by quinone molecules that are incorporated into the bilayer, such as ubiquinone (Figure 9).^132^, ^133^ This technique has been applied to study proteins ranging from the relatively small cytochrome bo3 from *Escherichia coli* ^*[*132]^ to the very large [NiFe]‐hydrogenase of *Ralstonia eutropha*.^133^, ^134^

#### 4.1.3 Long‐length conducting SAMs

As described in 3.1.1, slow electron transfer through long‐length alkanethiols (>9 carbon atoms) can introduce an artefact into biological electrochemistry experiments, with the limiting rate of the redox process reflecting the interfacial electrode‐to‐protein electron‐transfer rate instead of the speed of the biological reaction.^123^, ^124^, ^125^ This can be overcome by using more electrically conductive SAMs.^135^ For example, the use of a SAM containing a highly conjugated diarylethene moiety for modification of a gold electrode enabled fast electron transfer to the small blue copper protein azurin.^135^ The redox chemistry rate constant was higher (3–27 times faster) than obtained when using SAMs formed from alkanethiols of a similar length.^135^

Alternatively, redox‐active so‐called electron transfer “mediator” units can be built into SAMs. An example of such a conducting SAM precursor molecule is 1‐(10‐mercaptodecyl)‐1′‐benzyl‐4,4′‐bipyridinium dibromide, which was synthesized for immobilization of a H_2_‐producing [FeFe] hydrogenase.^136^ Unfortunately the enzymatic activity was only approximately 2.5 % of that expected based on solution‐state experiments, illustrating the complexity in optimizing such a SAM‐enzyme system.^136^

### 4.2 Aryl diazonium salt reduction

**Scheme 1:**
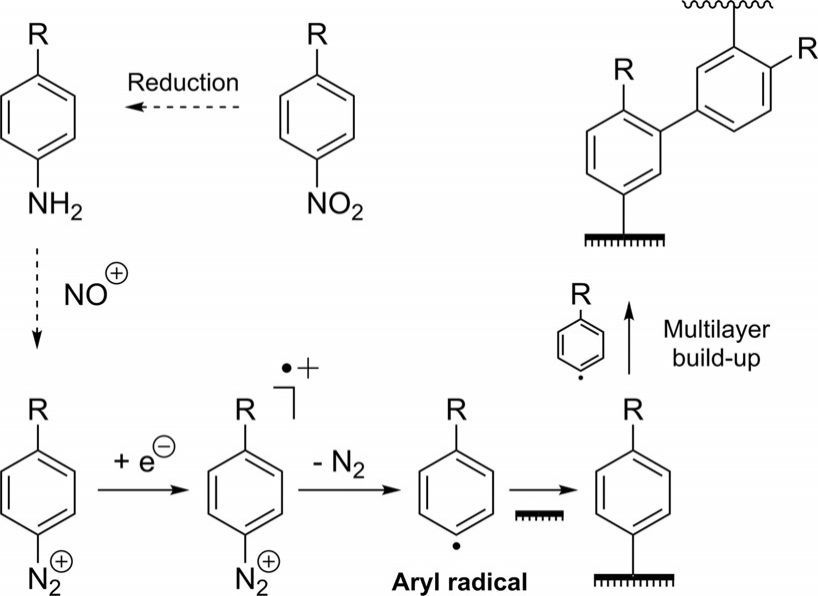
Electrochemical reduction of aryl diazonium salts resulting in the formation of a pacifying multilayer film.

**Figure 10:**
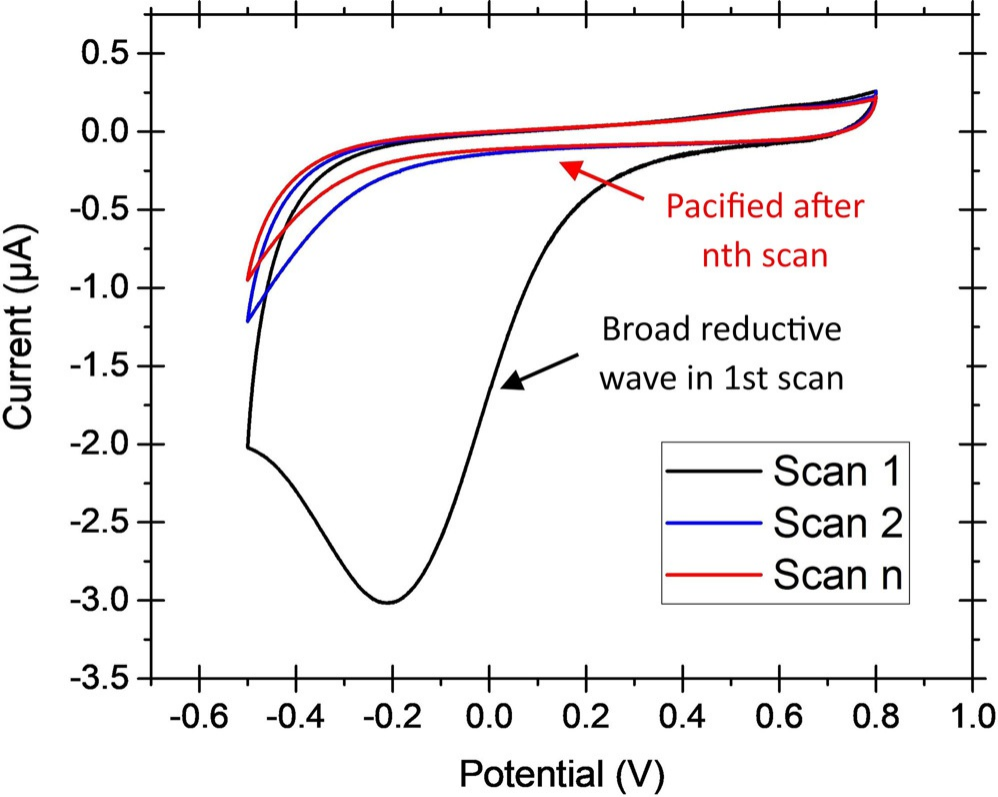
Characteristic cyclic voltammograms for the reduction of an aryl diazonium salt generated in situ from 2‐(4‐aminobenzyl)isoindoline‐1,3‐dione. Potential vs. Ag/AgCl (3 m KCl), scan rate 20 mV s^−1^, GC working electrode. Solvent system 1:5 water/acetonitrile+0.1 m Bu_4_NPF_6_+0.5 % *v*/*v* 6.6 m HCl. Ambient temperature.

The reduction of aryl diazonium salts for the functionalization of electrodes has been demonstrated on a variety of different materials including all conducting allotropes of carbon,^137^, ^138^, ^139^ silicon,^140^ ITO,^141^ and a range of metals including gold, platinum, and copper.^142^ A surface‐to‐carbon bond is formed via the one‐electron reductive formation of an aryl radical, which subsequently attacks the electrode surface, as illustrated in Scheme 1.^137^, ^143^, ^144^ Electrode functionalization using aryl diazonium salts is therefore electrochemically controllable (Figure 10), and can often be performed in aqueous or organic electrolyte.^137^, ^143^, ^144^ Either isolated aryl diazonium salts can be utilized, or they can be generated in situ using an aniline or nitrophenyl derivative and a source of the NO^+^ cation, such as NaNO_2_/HCl or NOBH_4_ (Scheme 1).^145^, ^146^

Given the range of commercially available aniline and nitrophenyl derivatives, the scope of chemical functionalities that can be introduced onto the surface using diazonium chemistry is comparable to that which can be accessed using commercial alkanethiol derivatives for SAM formation. Unlike SAM formation, this methodology is theoretically applicable to the covalent functionalization of any conducting surface, not just those that form a stable bond to sulfur. The redox stability of the electrode–carbon bond does not restrict the electrochemical window of biological experiments, and such surface modifications are also more amenable to long‐term storage than SAM‐modified gold surfaces.^144^, ^147^, ^148^, ^149^, ^150^

Diazonium electrode modification is not entirely without challenges. Multilayer formation can occur when further aryl radicals attack the unsaturated bonds of the aromatic π systems of the original monolayer, resulting in carbon−carbon bonds.^144^, ^151^, ^152^, ^153^ Alternatively, multilayers can arise from diazonium cations coupling to surface phenyl groups through azo bond formation.^144^, ^151^, ^152^, ^153^ Both modes of multilayer formation can contribute to the build‐up of an amorphous, organic, insulating layer on the surface of the electrode.^144^, ^151^, ^152^, ^153^ Methodologies to prevent or minimalize multilayer formation have been reported, such as the use and subsequent cleavage of bulky protecting groups,^154^ sterically hindering the 3,5‐positions of the aryl diazonium salt,^155^ and addition of the radical scavenger 2,2‐diphenyl‐1‐picrylhydrazyl (DPPH) to quench excess aryl radicals.^152^, ^153^

In the context of bioelectrochemistry, diazonium electrode modifications can be used to induce protein adsorption through non‐covalent interactions in a similar manner to that achieved using unmodified PGE or SAMs on gold. Table 1 summarizes some examples that have utilized different diazonium electrode functionalization methods. The flexibility of the method is illustrated by the literature precedence of the use of the same diazonium–protein immobilization strategy on a range of different electrode surfaces to immobilize a range of redox proteins. The coupling of dialdehydes to aryl amine groups, introduced through diazonium cation electrografting (Table 1, entry d) was used to immobilize several redox enzymes on both carbon^156^, ^157^ and gold^158^, ^159^ electrodes.

**Table 1:** Selected strategies for the functionalization of electrode surfaces through diazonium cation electrografting and subsequent chemical/electrochemical treatments that further modify the electrode surface polarity or provide chemical derivatives that can be exploited in covalent coupling strategies.

Entry	Surface functionalization strategy^[a]^	References
a)	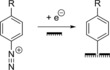	i) R=COOH^167^ ii) R=CH_2_COOH^168^ iii) R=CH=CHCOOH^169^ iv) R=NO_2_ 69, 170, 171, 172, 175 v) R=NH_2_ 176
b)	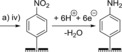	69, 170, 171, 172, 175
c)	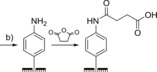	69
d)	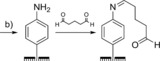	156, 157, 158, 159
e)	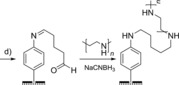	156
f)	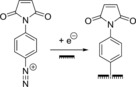	177

## 5 Covalent coupling of electrodes to native proteins

Attempts to physically adsorb proteins onto surfaces in an electroactive configuration are not always successful and, as described above, even when they do work, the adsorption strategies may be strongly dependent on the pH of the electrolyte solution. Alternatively, the film of molecules may only be transiently stabilized, with either misfolding^160^ or possible desorption processes leading to a steady decrease in redox activity. To avoid such problems the covalent attachment of proteins to electrode surfaces is desirable, particularly in biotechnological device development. Such a covalent coupling approach often requires a complementary surface functionalization strategy, so either thiol self‐assembly or diazonium modification is often used to introduce surface groups that will react with protein moieties.^154^, ^155^, ^156^, ^161^, ^162^, ^163^, ^164^

### 5.1 Peptide bond formation

**Figure 11:**
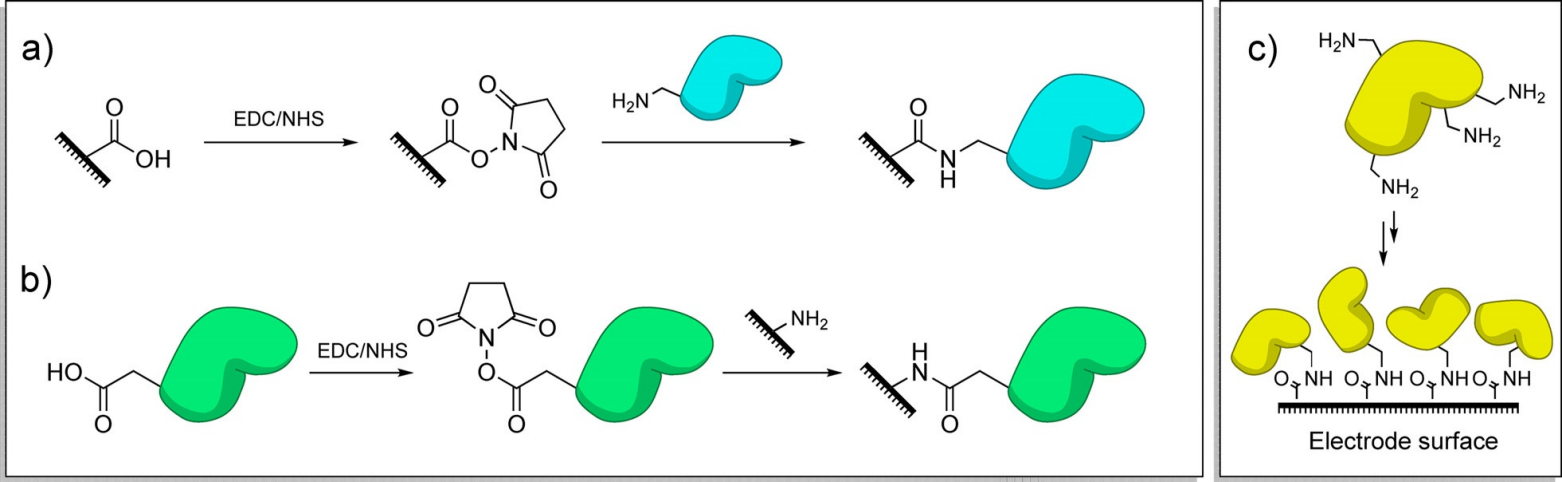
Amide bond formation between surfaces and protein residues, catalyzed through EDC/NHS activation. a) Activation of electrode‐surface carboxylic acid groups and reaction with protein lysine residues. b) Activation of carboxylic acid groups on the protein surface and reaction with electrode surface amine groups. c) Owing to the presence of many amine/carboxylic acid moieties on protein surfaces; immobilization through EDC/NHS activation often leads to a dispersion in immobilized protein orientation.

The most common covalent protein immobilization strategy is to mimic nature and generate peptide bonds, either through coupling carboxylic‐acid‐functionalized surfaces to protein‐surface lysine residues,^68^, ^154^, ^155^, ^156^, ^165^, ^166^, ^167^, ^168^, ^169^, ^170^ or crosslinking glutamate and aspartate residues that adorn protein surfaces to amine functionalized surfaces.^157^, ^170^, ^171^, ^172^, ^173^ The use of 1‐ethyl‐3‐(3‐dimethylaminopropyl)carbodiimide and *N*‐Hydroxysuccinimide (EDC/NHS) coupling is one way to form these peptide bonds (Figure 11).^166^, ^170^ Alternatives to EDC/NHS include EDC/sulfo‐NHS^174^ or *N*‐cyclohexyl‐*N*′‐(2‐morpholinoethyl)carbodiimide‐methyl‐*p*‐toluenesulfonate (CMC).^165^ Otherwise, long‐chain carboxylic‐acid‐terminated alkanethiols can be activated toward nucleophilic attack by an amine using trifluoroacetic acid anhydride through formation of interchain acid anhydrides.^163^

As described in Section 3.1.1, carboxylic acid or amine headgroups can be readily introduced onto gold electrodes through the selection of appropriate alkanethiols, and there are numerous examples of such a strategy being harnessed to form amide bonds to redox proteins.^162^, ^165^, ^166^ Likewise, carboxylic acid groups can be readily introduced onto electrode surfaces through the reduction of suitable diazonium precursors.^150^, ^167^, ^168^ Using isolated diazonium salts, the introduction of an amine functionality can be achieved through the reduction of the *p*‐aminodiazonium cation.^176^ Alternatively, the same surface modification can be achieved using the commercially available 4‐nitrobenzenediazonium tetrafluroborate salt, with electrochemical reduction being used to reduce the nitro “headgroups” into the desired amine functionalities in a post‐diazonium crosslinking step.^151^, ^158^, ^159^ The introduction of more reactive alkyl amine groups to electrode surfaces through diazonium modification can be achieved through the use of the 4‐aminoethylbenzenediazonium cation,^178^ or phthalimide‐protected alkylamine functionalities.^154^

Amide bond formation strategies have been used in the fabrication of many mediator‐free biosensors; EDC/NHS‐activated tyrosinase was crosslinked to aminophenyl groups on BDD electrodes and used to detect phenolic compounds.^170^ The EDC/NHS activation and crosslinking of horseradish peroxidase or cytochrome P450 enzymes to amine moieties on carbon electrodes has been used in the fabrication of biosensors for the detection of a series of pharmaceutically relevant drugs.^157^, ^171^, ^172^ Horseradish peroxidase could be used to sense levetiracetam,^171^ and the specific cytochrome P450 enzymes could be used to detect phenobarbital^157^ and codeine.^172^ The immobilization of an oxygen‐tolerant hydrogenase onto pyrene‐modified multiwalled carbon nanotubes coated onto PGE electrodes was also achieved through EDC/NHS coupling.^174^ The resultant derivatized PGE electrode was utilized as the anode in the fabrication of an enzyme H_2_/O_2_ fuel cell, which resulted in significantly improved current density and stability when compared to a fuel cell containing a hydrogenase electrode fabricated using simple adsorption procedures.^174^

The most significant limitation of such approaches is that regardless of whether carboxylic acid residues or lysine groups are targeted (multiple occurrences of such amino acid side chains on the surface of the redox protein or enzyme of interest are often present), significant dispersion in the orientation of the immobilized biomolecule commonly results (Figure 11). Careful genetic engineering of the target protein can overcome this problem. A recent publication by Lalaoui et al.^179^ reports the site specific immobilization of a laccase onto CNTs through the generation of a variant enzyme that only contains a single surface‐accessible lysine residue that is located proximal to the electron entry/exit type 1 copper center.^179^

### 5.2 Imine tethering

**Scheme 2:**
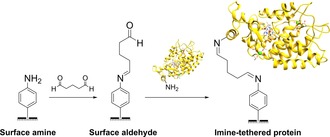
The use of an imine‐functionalized electrode to immobilize horseradish peroxidase, as detailed in reference 175.

Redox proteins or enzymes can also be covalently crosslinked to surfaces through imine bond formation between electrode‐surface aldehyde moieties and protein‐surface lysine residues (Scheme 2). For example, diazonium electrografting methods have been used to introduce aldehyde functionality onto electrodes (Table 1, entry d) that have subsequently been modified with enzymes, including acetylecholinesterase^156^, ^180^ horseradish peroxidase,^175^ and tyrosinase.^158^ Analogously, the reaction of glutaraldehyde with amine terminated SAMs yields an aldehyde‐functionalized surface that can be used to attach proteins through their surface lysine residues.^164^, ^181^, ^182^ To generate more stable covalent linkages, the imine bonds can be reduced to amine linkages using reagents such as sodium cyanoborohydride.^183^

As with amide bond formation between an electrode and surface**‐**lysine residues on a protein or enzyme, the same limitation remains; crosslinking electrodes to lysine residues that are not within close approach of the electron entry/exit site in a protein or enzyme will not yield electroactively bound biomolecules. Additionally, the presence of multiple surface lysine residues could result in dispersion in the orientation of the protein or enzyme on the electrode surface, as illustrated in Figure 11.

## 6 Crosslinking strategies for site‐specifically connecting proteins to electrodes

**Figure 12:**
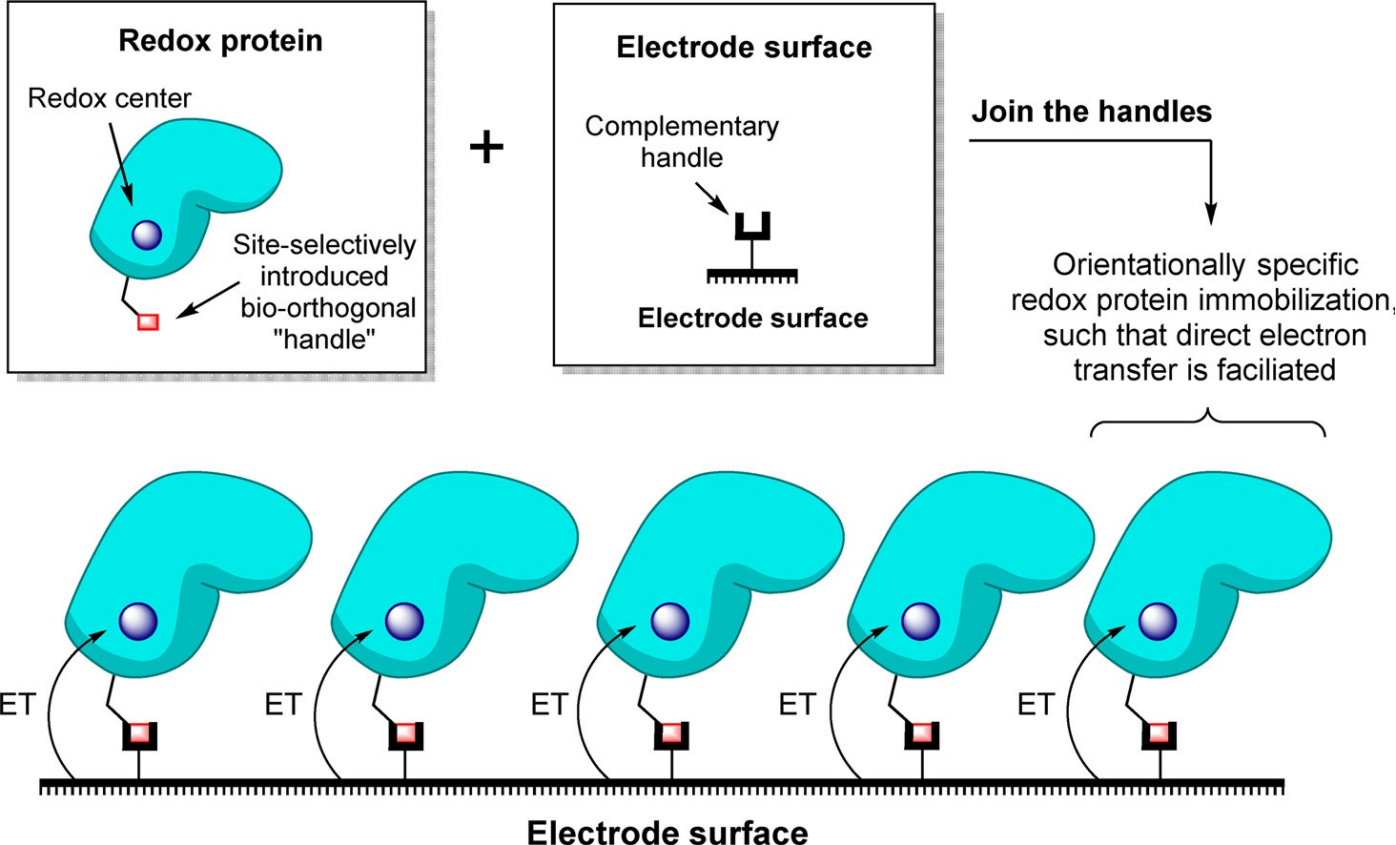
Generic strategy for site‐selectively crosslinking a redox protein to an electrode.

In theory, an excellent method to generate a uniform configuration of proteins or enzymes on a surface, with each biomolecule attached through the same single point, is to develop site‐selective covalent crosslinking strategies, as illustrated in Figure 12. This is often a complex process which usually requires a combination of genetic manipulation and surface chemistry to ensure that there is a single amino acid residue suitable for selective reaction with a complementary surface moiety. The advantage of modifying electrodes rather than non‐conductive solid substrates is that redox‐activated reactions such as diazonium salt electroreduction can be utilized in the surface chemistry (Section 3.2). However, this is tempered by the disadvantage that for direct electron transfer between the electrode surface and a redox protein or enzyme to be feasible the target amino acid reaction site must be sufficiently close to the electron entry/exit site (Section 2).

### 6.1 Redox‐center targeted binding

The easiest way to avoid the need for genetic manipulation of the target redox protein or enzyme is to devise an electrode binding strategy that anchors the biomolecule to the conducting surface through a non‐amino acid functionality. An obvious choice of center for such linking strategies is the electron entry/exit redox‐active cofactor of the protein/enzyme, since anchoring to the electrode surface through such a group will ensure that the biomolecule is crosslinked to the electrode in an electroactive configuration. We describe a number of approaches that have used this understanding of biological structure and function to rationally design bespoke wiring strategies for attaching proteins or enzymes to electrodes. The most obvious limitation of such cofactor‐targeted surface binding strategies is that biology utilizes a wide range of different redox‐active cofactors, as illustrated by Figure 1. Anchoring different classes of redox proteins or enzymes through a redox‐center‐targeted binding strategy therefore requires the design and optimization of many different chemical strategies: a non‐trivial synthetic task. In the case of enzymes such as lytic polysaccharide monooxygenases,^11^ the fact they contain a single redox site where the substrate must bind also introduces the challenge of whether linkers can be designed that do not hinder substrate binding and catalysis.

#### 6.1.1 Cofactor ligation

In some instances, redox‐active cofactors can be synthesized and incorporated into so‐called cofactor‐free “apo‐proteins.” This offers a route to generating redox proteins containing modified redox cofactors that have chemical functionalities complementary to those which can be added to the electrode surface. For example, incorporation of an azide‐functionalized heme group into cytochrome b562 enabled copper(I)‐catalyzed azide–alkyne cycloaddition to an alkyne‐functionalized CNT immobilized onto a GC electrode.^184^ Alternatively, for proteins containing metal‐electron entry/exit sites that have multiple ligands, genetic removal of an amino acid ligand residue offers the opportunity for structural reconstitution of the redox protein with an external ligand that is tethered to the electrode surface. This strategy has been demonstrated for an azurin variant.^188^ The copper‐coordinating histidine residue (His117) was replaced with a glycine residue through genetic manipulation.^188^ This opened up the coordination sphere around the redox‐active metal and allowed a pyridine headgroup tethered to an electrode surface to coordinate directly to the copper center, immobilizing the azurin in an orientation suitable for facile direct electron transfer and mimicking the native copper ligation that is afforded by His117.^188^ Surface attachment of the pyridine group was enabled by the synthesis of a thiol‐terminated linker that covalently bound to gold surfaces.^188^

#### 6.1.2 Substrate electrode tethers

**Figure 13:**
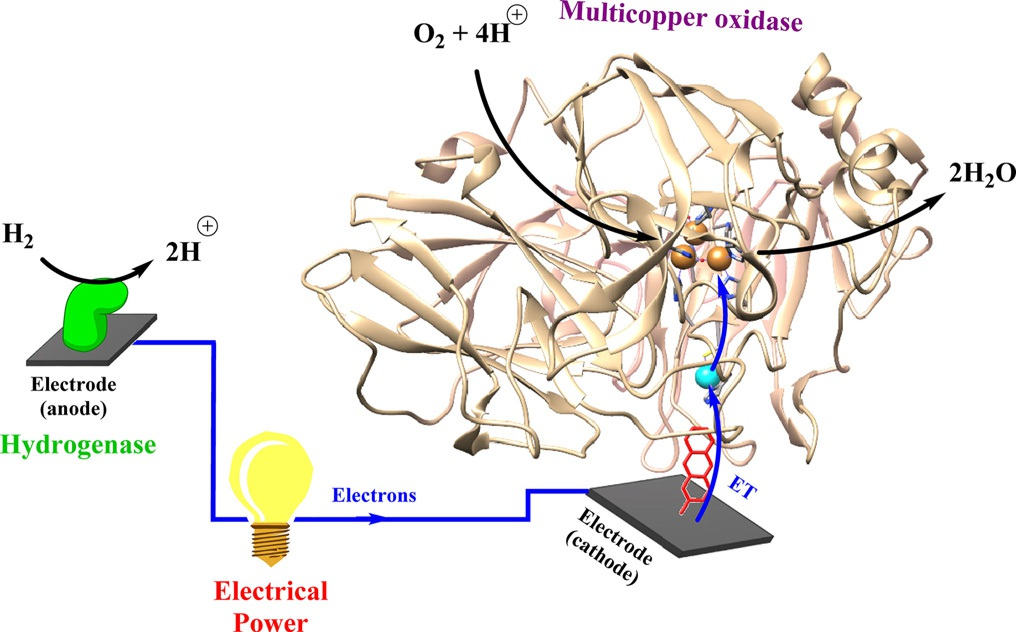
Membrane‐free H_2_/O_2_ fuels cells can be fabricated by coupling the redox activity of hydrogenases to oxidases.^185^, ^186^, ^187^ The orientation of the multicopper oxidase Trametes versicolor laccase III (PDB code: 1KYA) onto an electrode surface for O_2_‐reduction catalysis can be achieved through the modification of the electrode surface with anthracene substrate mimics, thereby anchoring the enzyme by the binding pocket and allowing facile direct electron transfer.^185^, ^186^

With redox enzymes that internally transfer electrons from the oxidation of a substrate in one binding pocket to reduce a second substrate in a second binding site, surface attachment can be achieved based on the “lock and key” model^189^ of site‐selective enzyme–substrate binding. For example, multicopper oxidases (that have evolved to couple organic‐substrate oxidation at one copper site to oxygen reduction at another copper site) can be immobilized for use as Pt‐free, low‐overpotential O_2_‐reduction electrocatalysts through the use of surface‐attached organic‐substrate mimics (Figure 13).^185^, ^186^ The highly conjugated nature of an anthracene electrode linker, immobilized onto graphite using diazonium chemistry, was shown to ensure rapid electron transfer from the electrode surface to laccase.^185^, ^186^ Using a similar strategy, surface naphthoic acid moieties were effective in the immobilization of bilirubin oxidase from *Myrothecium verrucaria*.^190^ O_2_ reduction by this enzyme was externally wired to a hydrogenase‐coated electrode to construct an all‐enzyme, membrane‐free H_2_/O_2_ fuel cell where H_2_‐oxidation is used as the source of electrons for O_2_ reduction (Figure 13).^187^

In a similar vein, the surface binding of DNA is used to immobilize redox proteins for the electrochemical interrogation of the redox reactions that may underpin DNA translation and repair in vivo.^191^

### 6.2 Cysteine‐based surface ligation

As the sole thiol‐containing canonical amino acid, cysteine presents a unique chemical functionality that can be harnessed in the design of biochemical ligation methodologies that selectively target only cysteine residues.^192^, ^193^, ^194^, ^195^ This chemical selectivity is complemented by the fact that, relative to other amino acids, cysteines are rarely present on protein surfaces.^196^ Thus, it can be relatively trivial to use site‐directed mutagenesis and chemical biology conjugation methods to engineer proteins and enzymes with single, covalently modified surface‐cysteine residues^192^, ^193^, ^194^, ^195^ Such strategies are of enormous value in the development of new biopharmaceutical therapies.^192^, ^195^ Surface‐attachment strategies have been developed along similar lines, with the added consideration that for direct electron transfer between a conducting surface and a redox protein or enzyme, the cysteine residue must serve as a tethering site that holds the redox protein/enzyme in an electroactive orientation.^43^, ^89^, ^197^, ^198^

The most significant limitations to the use of cysteine residues for enzyme electrode “wiring” applications arise from the potential for these residues to form intermolecular disulfide bonds,^199^ or to cause misfolding through the formation of non‐native disulfide bond formation,^200^ or through the accidental introduction of an extra metal–ligand residue to a metalloprotein or enzyme. For example, iron‐sulfur cluster incorporation into a protein structure is dependent on metal cluster binding to a highly conserved sequence of cysteines,^1^ and addition of extra residues can be used to convert a [Fe_3_S_4_] center into [Fe_4_S_4_].^201^ To avoid the issue of unwanted disulfide bond formation, proteins displaying free surface‐cysteine residues can be kept under reducing conditions through addition of dithiothreitol (DTT). However, because DTT contains thiol groups, this reducing agent must be removed before surface bioconjugation is attempted, to avoid unwanted reactions between DTT and the electrode surface.^202^

#### 6.2.1 Direct immobilization onto gold

As described in Section 3.1, the formation of SAMs onto gold electrodes is facilitated by the generation of gold–sulfur bonds. An analogous approach is to therefore graft surface‐cysteine‐containing proteins or enzymes directly onto gold surfaces.^203^, ^204^ The ability of this approach to immobilize redox proteins in chosen orientations has been definitively demonstrated using a cytochrome b 562 engineered to present cysteine residues on either the long axis or short axis.^203^ The resultant orthogonal orientations of these different protein variants on atomically flat gold was then observed using STM imaging.^203^ As with SAMs on gold, a significant limitation of this method is the redox instability of such covalent electrode–protein modifications (Section 3.1). The fact that electroreduction can be used to break Au−S bonds at relatively high reducing potentials impedes the use of this methodology for studying important biofuel reactions such as hydrogen production.

#### 6.2.2 Thiol–Michael addition click reactions

**Scheme 3:**
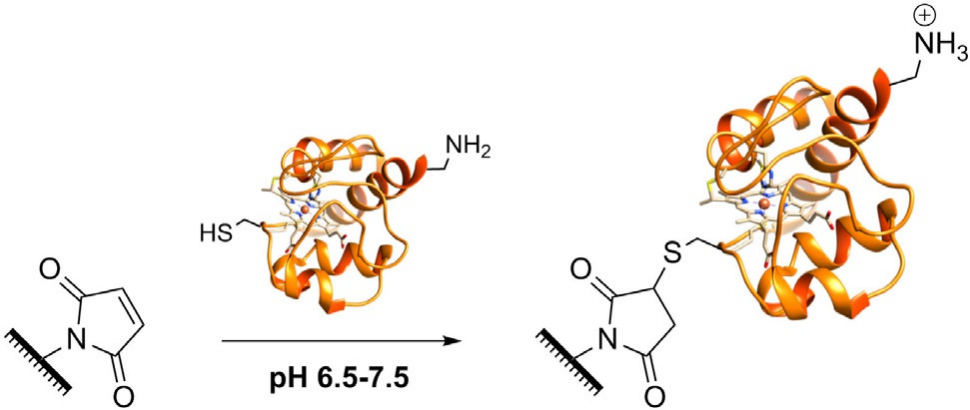
Maleimide‐thiol Michael addition reactions between maleimide groups introduced onto an electrode surface and cytochrome c surface cysteine residues.^177^

In recent years, the reaction between cysteine residues and unsaturated π systems through Michael addition has been used as a general tool for the chemical modification of many proteins, extending well beyond electrode–protein‐surface ligation strategies.^205^, ^206^, ^207^, ^208^ Numerous different methodologies using different π systems have been optimized for different applications.^205^ In the field of electrode ligation, surfaces have been functionalized with maleimide groups, the most reactive of the commonly available vinyl Michael acceptors.^205^ Between pH 6.5 and 7.5, maleimide groups react selectively with thiols, as within this pH range amines remain protonated and are thus not of a high enough nucleophilicity to partake in competing side reactions (Scheme 3).^198^

Maleimide groups can be introduced onto electrode surfaces using a variety of techniques, including the use of specially designed alkanethiol SAMs,^206^, ^209^ diazonium cation electrografting (Table 1, entry f),^177^ and sequential electrochemical and solid‐phase preparation.^198^ The reaction between surface‐maleimide groups and one of the two thiol groups that naturally occur near the heme cofactor of cytochrome c results in the immobilization of this protein in a near‐site‐specific orientation that is suitable for direct electron transfer (Scheme 3).^177^

### 6.3 Crosslinking to unnatural amino acids

Unnatural amino acid (UAA) mutagenesis is a technique which utilizes codon reassignment to expand the amino acids available for the synthesis of a target protein of interest.^204^, ^205^, ^206^, ^210^, ^211^, ^212^, ^213^, ^214^, ^215^ This allows amino acids with novel functionalities to be introduced at specific locations within proteins, and such residues can be subsequently targeted in bio‐orthogonal chemical ligations.^194^, ^214^, ^215^ This is a rapidly expanding field of research^204^, ^205^, ^206^, ^210^, ^211^, ^212^, ^213^, ^214^, ^215^ and there are now examples of such methodologies being adapted for covalent crosslinking of redox proteins and enzymes to electrode surfaces.^216^, ^217^, ^218^ A practical consideration that makes the use of UAA mutagenesis potentially unsuitable for “wiring” redox metalloenzymes onto electrodes is that highly complex biosynthetic pathways can make protein overexpression challenging; in such scenarios substantial quantities of synthetic UAA may be required to generate useable quantities of UAA containing protein.

**Figure 14:**
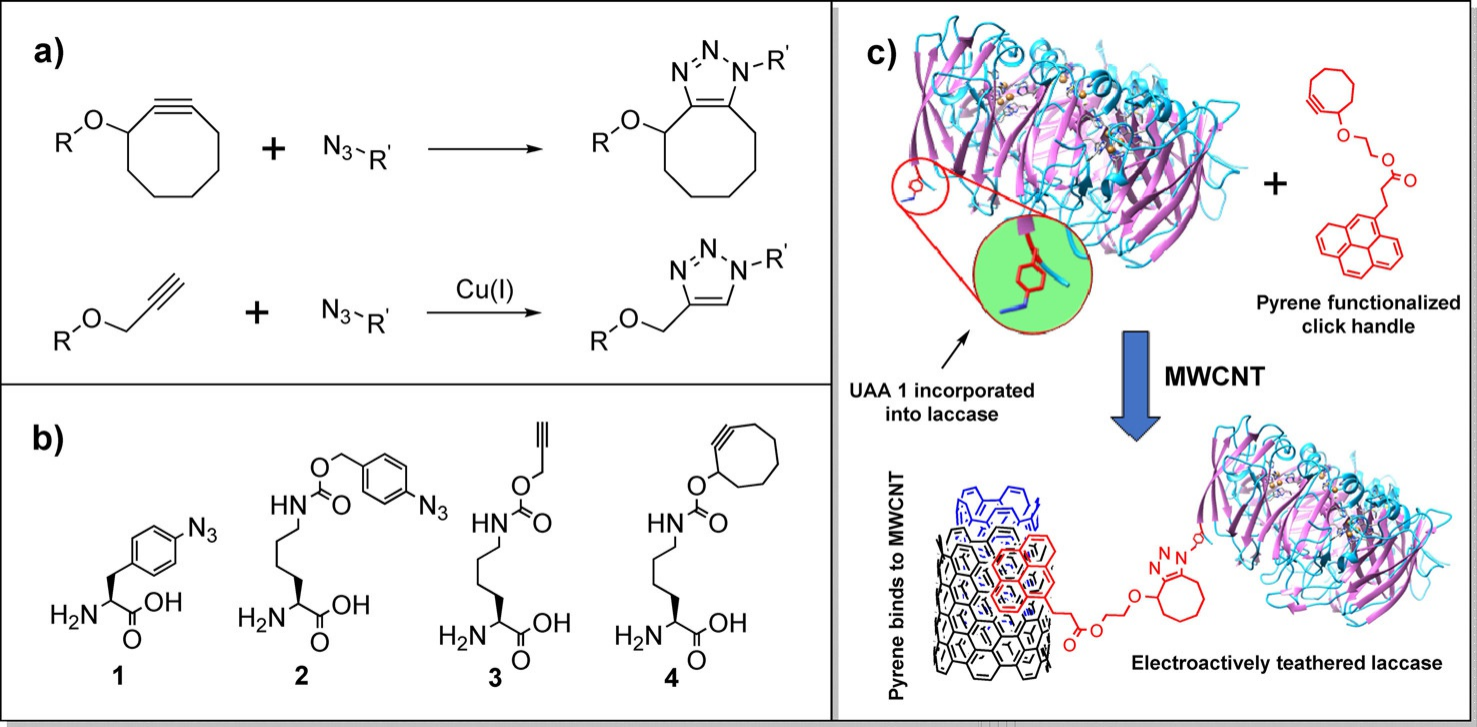
Azide–alkyne cycloaddition click reactions between surfaces and proteins. a) Top: copper‐free non‐catalyzed azide–alkyne cycloaddition click reaction, promoted by a ring‐strained alkyne. Bottom: copper‐catalyzed azide–alkyne cycloaddition reaction. b) Owing to the precedence for unnatural amino acids bearing both alkyne and azide functionalities, it is possible to functionalize a protein with either azides or alkynes. c) The site‐selective electroactive immobilization of a laccase onto a MWCNT using a copper‐free non‐catalyzed azide–alkyne cycloaddition click reaction between an azide‐functionalized UAA and a surface‐confined cyclooctyne, as described in ref. 216. Residues from only one monomer are depicted (PDB ID: 3CG8^222^).

For UAA mutagenesis to serve as an immobilization methodology, functionalities complementary to those of the UAA must also be introduced to the electrode surface. Azide–alkyne cycloaddition click reactions therefore represent an attractive approach since methods for the introduction of these moieties onto electrode surfaces have been developed for other applications such as DNA sensor development.^219^ Should the use of a copper catalyst for activation of the cycloaddition reaction be undesirable, copper‐free reactions can be performed through the use of ring‐strained alkynes (Figure 14).^220^ Surprisingly, based on the robust nature of this chemical ligation strategy,^221^ the only known example using azide–alkyne UAA reactions for the site‐specific linkage of a redox protein or enzyme to an electrode is the immobilization of the 4‐azido‐l‐phenylalanine (**1**, Figure 14) containing laccase from *Streptomyces coelicolor* onto a MWCNT‐coated electrode functionalized with complementary cyclooctyne containing linkers (Figure 14).^216^ Interestingly, the most effective orientation for direct electron transfer was found to be one that tethered the laccase at a site distal from any redox centers but adjacent to a water channel; the structured water molecules are thought to substantially enhance the electron transfer rate between the electrode and the laccase.^216^ A similar strategy has also been used to “wire” whole bacteria to electrodes.^217^ Through the incorporation of UAA **1** into an alcohol dehydrogenase that is displayed on the surface of *E. coli*,^217^ copper(I)‐catalyzed cycloaddition to an alkyne functionalized SAM linker was used to bond bacterial cells to a gold surface.^217^

**Figure 15:**
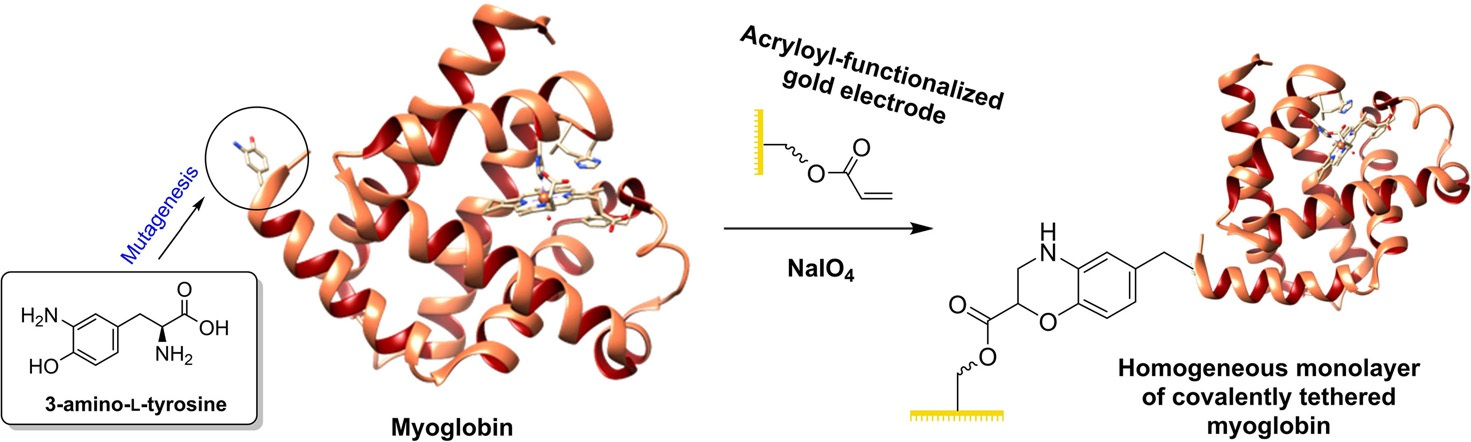
Covalent crosslinking of myoglobin to a gold electrode as a homogeneous monolayer, through the incorporation and Diels–Alder reaction of a 3‐amino‐l‐tyrosine residue.^218^

Other, non‐azide–alkyne chemical ligation strategies can be realized through the use of different UAA residues. The incorporation of 3‐amino‐l‐tyrosine (NH_2_Tyr) into myoglobin has been used to covalently attach the protein onto a gold surface derivatized with acryloyl moieties, courtesy of a Diels–Alder reaction specific to NH_2_Tyr (Figure 15).^218^ The rate of electron transfer between the electrode and myoglobin was slow, which was attributed to the length of the anchoring tether (26.7 Å).^218^

## 7 Summary and outlook

Although highly informative reviews have been written on the powerful bio‐analysis technique of protein film electrochemistry, the fundamental step of protein film formation is often overlooked. This is understandable; to establish the technique of PFE it has been necessary to provide substantial insight into electrochemical method design and data analysis approaches, as well as showcase the powerful insight which can be gained from conducting such experiments on biologically and biotechnologically important systems. To complement such papers, this review aims provide an up to date and broad ranging overview of the many different surfaces, surface modification strategies and protein conjugation approaches which can be used to “wire” redox‐active macro‐biomolecules to electrodes.

Owing to the diversity of redox proteins and the wide range of possible usages, there are currently no universal surface‐confinement approaches. By comparing and critiquing the different approaches we hope to provide the reader with a one‐stop reference library that will aid selection of appropriate electroactive surface immobilization techniques for use in studying/harnessing new redox proteins and enzymes, or introduce them to this diverse field. For gifted bio‐conjugation chemists, we hope to inspire the need for further method development, having emphasized that we still lack a way to generate robust, site‐selective bonds from any protein or enzyme to any electrode.

## Conflict of interest

The authors declare no conflict of interest.

## Biographical Information

*Nicholas Yates received his M.Chem. degree in Chemistry from the Department of Chemistry at The University of York in 2016. He is currently engaged in Ph.D. research at the University of York, developing new methods of bio‐orthogonal site‐selective protein immobilization under the supervision of Dr. Alison Parkin and Dr. Martin Fascione*.

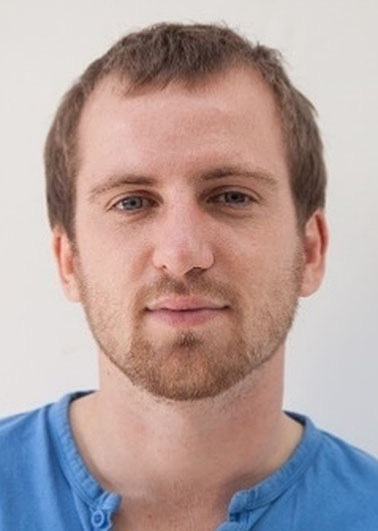

## Biographical Information

*Dr Martin Fascione received his Ph.D. from the University of Leeds in 2009, working under the tutelage of W. Bruce Turnbull on the stereoselective synthesis of 1,2‐cis‐glycosides. Following a post‐doctoral period in Leeds, he was then awarded a Marie Curie International Outgoing Fellowship to study the mechanisms of carbohydrate‐processing enzymes with Professor Steve Withers, FRS, at the University of British Columbia in Vancouver, Canada (2012–2013) and Professor Gideon Davies, FRS, FMedSci, at the University of York, (2013–2014). In August 2014 he took up a lectureship in the York Structural Biology Laboratory within the Department of Chemistry. His research interests include chemical biology, chemical/enzymatic modification of proteins and synthetic carbohydrate chemistry*.

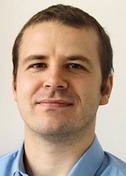

## Biographical Information

*Dr. Alison Parkin received her M.Chem. (2004) and D.Phil. in Chemistry (2008) from the University of Oxford where Professor Fraser Armstrong, FRS, inspired her passion for redox biology. She was a Junior Research Fellow at Merton College, Oxford from 2008–2012, and then joined the University of York as the Anniversary Research Lecturer in Chemistry. Her research group is focused on harnessing electrochemistry as an analytical and technological tool for probing and controlling chemical reactions, particularly those that occur in living systems. Alison was awarded an Early Career Research Award in 2013 by the Biochemical Society*.